# Authors’ Response to Peer Reviews of “Impact of the COVID-19 Pandemic on Routine Childhood Vaccination Coverage in Ecuador From 2019 to 2021: Comparative Analysis”

**DOI:** 10.2196/84851

**Published:** 2025-10-17

**Authors:** Jose Sanchez, Alejandro Arjuna Rodriguez Sr, Kimberlly Pamela Montenegro Cuello Sr

**Affiliations:** 1 Faculty of Health Sciences and Human Well-being, Universidad Indoamérica, Avenida Machala y Sabanilla, La Pradera, Quito, 170509, Ecuador, 593 0984663224; 2 Faculty of Health Sciences "Eugenio Espejo", Universidad UTE, Quito, Ecuador

**Keywords:** COVID-19 pandemic, vaccination coverage, Ecuador, immunization, routine vaccination, health disparities, vaccine hesitancy

*This is the authors' response to peer review reports related to “Impact of the COVID-19 Pandemic on Routine Childhood Vaccination Coverage in Ecuador From 2019 to 2021: Comparative Analysis.”*

## Round 1 Review

### Reviewer G [1]

#### Major Comments

*1. Please complete the manuscript [2] by adding the results and interpretation of the Joinpoint regression analyses. The authors claimed that Joinpoint regression analyses were conducted but did not present and discuss the results. More importantly, please note that the Joinpoint analysis requires at least 7 time points. The authors only included 3 time points (2019, 2020, 2021). I suggest either calculating vaccination coverage percentages for at least 7 years to run the Joinpoint analysis or just presenting the descriptive statistics for each year without using the Joinpoint analysis.*

**Response:** We have removed all references to Joinpoint regression analysis from the manuscript. We acknowledge that our study has only 3 time points (2019, 2020, 2021), which is insufficient for Joinpoint analysis. The Methods section now clearly states that we used descriptive statistics and comparative analysis appropriate for our data structure.

*2. There is a figure that plots the vaccination coverage rates in 2019, 2020, and 2021, but the authors did not provide any description or interpretation of the figure.*

**Response:** We have added comprehensive descriptions for all figures. For example: "Figure 1 illustrates the temporal trends in vaccination coverage for key vaccines from 2019 to 2021. The visualization clearly demonstrates the progressive decline in coverage rates, with the most dramatic decreases occurring between 2020 and 2021.”

*3. Please add descriptions for all tables and figures.*

**Response:** Complete descriptions have been added for both tables and all figures, explaining their content and relevance to the study findings.

*4. Please be more specific in the Data Analysis section; for example, please clearly mention what was meant by trend analysis and comparative analysis and include any specific descriptive summaries and/or statistical tests you used.*

**Response:** The data analysis section has been expanded to specify: “We calculated absolute and relative changes in coverage between time periods” and “Coverage data were plotted over time to visualize trends and identify patterns of decline or recovery across different vaccines and regions using the *matplotlib* and *seaborn* libraries in Python.”

*5. Please improve the organization of the Results section. For example, the regional disparities were discussed at the end of the section, yet they were presented in the first table.*

**Response:** The Results section has been reorganized to present (1) overall vaccination coverage trends, (2) vaccine-specific coverage analysis, and (3) regional and provincial disparities, maintaining logical flow and consistency with table presentation.

*6. Please narrow the focus of the manuscript. It seems that the authors aim to characterize the changes in routine childhood vaccination before and after the COVID pandemic, but in the manuscript, the authors also mention the disparities in getting the COVID-19 vaccine among the entire Ecuador population. These seem like relatively separate topics and could possibly be studied in two manuscripts.*

**Response:** We have removed all references to COVID-19 vaccination coverage in the general population and focused exclusively on routine childhood vaccination, as suggested. The manuscript now maintains a clear, unified focus.

*7. Please support all claims with data or citations. For example, if the authors decide to also study the disparities in COVID-19 vaccine access, please include relevant data analysis results in the manuscript.*

#### Minor Comments

*8. At the start of the Data Analysis section, please cite the specific software used.*

**Response:** We added the following: “Statistical analyses were performed using SPSS (version 28.0; IBM Corp). Trend visualization was performed using the *matplotlib* and *seaborn* libraries in Python.”

*9. I was wondering if there is data from after the pandemic (2022 and beyond), so the authors can examine whether routine childhood vaccination coverage went back up or kept declining.*

**Response:** We have added the following to the Limitations section: “The analysis is limited to 2019‐2021, preventing assessment of recovery efforts that may have begun in 2022‐2023.” We noted this as an important area for future research.

10. *Please clarify what Table 1 presents and why it is included.*

**Response:** We added an explanation: “Table 1 presents population data across Ecuador’s 4 main regions and 24 provinces, providing context for understanding vaccination disparities and calculating coverage rates.”

### Reviewer L [3]

#### Major Comments

*1. Clarity on methodology: The study uses observational comparative analysis and descriptive statistics but would benefit from additional details on the specific statistical tests used (eg, Joinpoint regression) and any confidence intervals or measures of significance included.*

**Response:** We have expanded the Methods section to clarify: “We calculated absolute and relative changes in coverage between time periods using appropriate descriptive statistics. Coverage rates were calculated following World Health Organization guidelines for vaccination coverage assessment.”

*2. Policy and programmatic implications: While the discussion clearly outlines the negative impact on vaccination coverage, the manuscript could be strengthened by offering more specific recommendations for public health policy, especially regarding catch-up campaigns or digital infrastructure improvements to track immunization.*

**Response:** We have significantly expanded the Policy Recommendations section, including targeted catch-up vaccination campaigns, health system strengthening, community engagement strategies, digital health innovations, and integrated service delivery models.

*3. Sociodemographic context: The analysis highlights disparities but could be improved by integrating more granular sociodemographic information (eg, income, ethnicity, rurality) to provide a deeper understanding of inequities in coverage and guide targeted interventions.*

**Response:** We have enhanced the discussion of disparities and added to the Limitations: “Detailed individual-level socioeconomic data were not available, limiting the ability to fully analyze equity impacts.” We also expanded the discussion of rural/urban and Indigenous population impacts.

#### Minor Comments

*4. Language and style: The manuscript would benefit from light editing to improve flow and reduce minor typographical and grammatical errors.*

**Response:** The entire manuscript has been thoroughly edited for language, flow, and typographical errors.

*5. Figure/table integration: Tables are rich in data, but would be more useful if the text referenced key figures and included short interpretation notes to help readers navigate large data points.*

**Response:** We have improved integration between the text and tables/figures with specific references—for example, “Table 2 presents comprehensive coverage data showing this concerning trend”—and added interpretative notes throughout.

*6. Redundancy in the Introduction: Some repetition in the early paragraphs could be streamlined to maintain reader engagement.*

**Response:** We have eliminated redundancies in the Introduction and improved flow between paragraphs.

### Reviewer M [4]

#### Major Comments

*1. The tables and figures should be well-labeled and referenced.*

**Response:** All tables and figures now have clear, descriptive titles and are properly referenced throughout the text with specific callouts and interpretations.

*2. The limitations of the study are briefly mentioned.*

**Response:** We have significantly expanded the Limitations section to include temporal scope limitations, the lack of individual-level socioeconomic data, causal attribution challenges, subnational granularity issues, and reliance on administrative data.

*3. The statistical methods should be well-presented.*

**Response:** The Data Analysis section has been expanded with specific details about the software used (SPSS 28.0), analytical approaches (descriptive statistics, comparative analysis, geographical analysis), and visualization methods (Python libraries).

#### Minor Comments

*4. The Methods should be more explanatory.*

**Response:** The Methods section has been substantially expanded to include detailed descriptions of the study design rationale, data source specifications, study population definitions, vaccination coverage metrics, ethical considerations, and data quality measures.
